# Sclerotia-Mediated Soil Microbiome Modulation in Rice–Rapeseed Cropping Systems

**DOI:** 10.3390/jof11100755

**Published:** 2025-10-21

**Authors:** Mirza Abid Mehmood, Jianguang Wang, Jiasen Cheng, Jiatao Xie, Daohong Jiang, Yanping Fu

**Affiliations:** 1 The Provincial Key Lab of Plant Pathology of Hubei Province, College of Plant Science and Technology, Huazhong Agricultural University, Wuhan 430070, China; 2 National Key Laboratory of Agricultural Microbiology, Huazhong Agricultural University, Wuhan 430070, China; 3 State Key Laboratory of Vegetation Structure, Function and Construction, School of Ecology and Environmental Science, Yunnan University, Kunming 650500, China; jgwang@ynu.edu.cn

**Keywords:** sclerotia, cropping system, oversummering, overwintering, microbial diversity

## Abstract

*Rhizoctonia solani* (Rs) and *Sclerotinia sclerotiorum* (Ss) are devastating pathogens of rice and rapeseed, contributing 20–69% and 10–50% of yield losses, respectively. These pathogens develop resistant overwintering and/or oversummering sclerotia, which serve as inocula for infection in the subsequent season under favorable conditions. The present study was designed to investigate the month-wise variation in microbial diversity by mixing Rs and Ss sclerotia separately in rice-rapeseed rotation field soil, thereby identifying key microbial players associated with specific sclerotia and their implications for subsequent crops. Therefore, we incubated 2.5 g of Rs and Ss sclerotia in 100 g of soil for 3 months to mimic the field conditions and subjected month-wise soil samples to 16S rRNA and ITS2 sequencing. Data analysis of bacterial communities revealed diversity, richness, and evenness in Ss treated soil samples compared to the control, while fungal communities exhibited less diversity. These results were also evident in PCoA and hierarchical clustering, where control and treated samples were scattered in 16S rRNA and ITS sequencing. Genus level diversity exhibited enrichment of bacterial genera with known beneficial potential, notably *Acidibacter*, *Stenotrophobacter*, *Sphingomonas*, *Flavisolibacter*, *Gaiella*, and *Neobacillus* in control. Beneficial bacterial genera such as *Ramlibacter*, *Geomonas*, *Kofleria*, *Nitrospira*, and *Paraflavitalea* were enriched in Ss treated soil samples. The addition of Ss and Rs sclerotia activated several beneficial fungi, notably *Trichoderma*, *Talaromyces*, *Clonostachys* in Ss treated samples, and *Vermispora*, *Hyalorbilia*, *Mortierella*, *Lecanicillium* in Rs treated samples. Additionally, Rs treated soil samples also activated pathogenic genera, including *Typhula*, *Fusarium*, and *Rhizoctonia*. Sclerotia in soil modulates the microbiome and activates beneficial and pathogenic microbes. During the off-season, the *Sclerotinia* inoculum pressure in the soil reduces, and it is safe to grow crops next season. Whereas, in the case of *Rhizoctonia* infected soil, it is suggested to avoid growing crops susceptible to wilt, root rot, and blight. However, field experiments to understand the pathogen–pathogen interactions around the sclerotiosphere require further exploration.

## 1. Introduction

Sclerotia are thick, resistant off-season survival structures produced by several pathogenic fungal species, including *Rhizocotnia solani* (Rs) Kühn and *Sclerotinia sclerotiorum* (Ss) (Lib.) de Bary, causing rice sheath blight and stem rot, respectively [1,2,3]. Rs and Ss are necrotrophic fungi have broad host ranges of more than 250 and 400 plant species, respectively [2,4,5,6]. Previous studies claimed that sclerotia remain viable for 3–5 years, but recent findings confirmed that sclerotia can survive even after 30 years at −3.5 °C in permafrost conditions [7,8,9,10]. Earlier studies observed that Rs sclerotia can withstand extreme stresses, including placing them in the desiccator and immersing them in sterile water and paddy soil, and survive for more than 10 months [11]. The strong survival ability of sclerotia is due to the complex mixture of proximates and minerals that help them withstand the harsh environment [12,13,14,15]. Rs is considered the second most notorious pathogen of rice, resulting in substantial global yield losses. It has caused yield losses of up to 20–69% in temperate and tropical regions [16]. The disease area affected by rice sheath blight in China is 15–20 Mham^2^ annually, with yield losses up to 10–50% during the epidemic years along the Yangtze River in South China [17,18,19,20,21]. Severely infected paddy field soil yielded 73–636 Rs sclerotia from one liter soil samples obtained from 0 to 7.6 cm depth [22]. In our earlier study, we reported that the abundance of Rs sclerotia increased after 3 months of incubation under controlled conditions [23].

Similarly, Ss accounted for 81% of total disease related losses in rapeseed from 2007 to 2016 [21]. Different studies reported various inoculum densities required for successful infection, but it was believed that 3.2 g sclerotia/m^2^ of kidney bean field resulted in 95% stem rot infection in rapeseed [24]. Another study on dry beans confirmed that 0.2 g sclerotia/kg of soil caused moderate to severe losses [25]. In a subsequent study on inoculum density, Adam and Ayers found 160–820 sclerotia of *S. minor* and *S. sclerotiorum*/kg of natural soil [26]. Later, the infection of Ss on lettuce fields produced 2.9 sclerotia/ 100 g of soil in Yuma County [27]. Recently, Taylor et al. [28] confirmed that 1.6–3.0 g of sclerotia can be produced by a single infected oilseed rape or lettuce plant. On the contrary, the population of Ss diminished in rapeseed–fallow soil samples amended with Ss sclerotia compared to the control, as confirmed using next generation sequencing [29].

Organic matter amendment in the soil positively impacts plant health and yield by providing essential nutrients. These nutrients influence microbial communities, which help reduce the effects of soilborne pathogens by depicting suppressive effects [30,31,32,33]. Several studies reported that beneficial microbes with biocontrol activity parasitize Ss sclerotia within the soil, including *Trichoderma virens*, *Bacillus subtilis*, *Sporidesmium sclerotivorum*, *Coniothyrium minitans*, *Streptomyces lydicus*, *Pythium oligandrum*, *Trichoderma* spp., and *Talaromyces flavus.* The abundance of beneficial microbes is affected significantly when sclerotia are mixed with the soil as well as serve as a bait for microbes with biocontrol potential [29,34,35,36,37]. Shrestha et al. [38] concluded that the addition of *Trichoderma harzianum*, *T. asperellum*, and *Streprtomyces griseovirdis* separately and in combination along with anaerobic soil disinfestation did not reduce the germination of *S. rolfsii* sclerotia. Wang et al. [39] revealed that the incubation of Ss sclerotia at 35 °C and low oxygen levels resulted in 100% death, and the relative abundance of *Talaromyces* and *Bacillus* was higher in the high temperature treatments [29]. While another study on soil samples amended with Rs sclerotia revealed enrichment of beneficial microbes with potential roles in the nitrogen cycle and plant growth promotion [23].

The individual effects of Rs and Ss sclerotia on their respective hosts are well-elaborated. However, the temporal dynamics of soil microbiome in response to these sclerotial amendments within the context of rice–rapeseed rotation remain unexplored. Information regarding the effect of different concentrations of Rs sclerotia amendment on soil bacterial diversity has been documented [23], but data on fungal diversity using culture-independent approaches are scarce. The rice-rapeseed rotation is a commonly used cropping system in South China. Rs sclerotia produced on rice must survive in the soil after crop harvest, where soil water content drops to 60–70% for cultivation of rapeseed. In contrast, Ss sclerotia must face a hot summer in the rapeseed–fallow field or waterlogged conditions due to paddy cultivation. These contrasting pressures impose unique selection on microbiomes associated with different pathogenic sclerotia. Therefore, we hypothesized that amending soil with Rs and Ss sclerotia would activate similar microbial consortia due to their structural and compositional similarity in the absence of the host plant, mimicking field conditions. The present study was designed to investigate the month-wise variation in microbial diversity by mixing Rs and Ss sclerotia separately in rice-rapeseed rotation field soil, thereby identifying key microbial players associated with specific sclerotia and their implications for subsequent crops.

## 2. Materials and Methods

### 2.1. Preparation of Soil and Sclerotia

Soil samples were collected from five different spots at a depth of 0–15 cm in a rapeseed–rice rotation field in Shayang County, Hubei Province, China. The pH of the soil was 6.78, total nitrogen and carbon contents were 0.20% and 1.66%, respectively [23]. To create a composite soil sample, the soil was allowed to dry at room temperature, followed by thorough mixing of representative soil samples. Soil was cleaned from roots with forceps and sieved through a 2 mm mesh, as stated earlier, without adding or removing nutrients [40]. Peeled, sliced, and diced potatoes and carrots were autoclaved for one hour at 121 °C in 500 mL separate conical flasks, to produce sclerotia of the model strains of *R. solani* (Rs) WH-1 strain and *S. sclerotiorum* (Ss) strain 1980 [41]. After sterilization, 3–4 agar plugs of actively growing mycelia of Rs and Ss were shifted to the sterilized flasks followed by incubation at 28 ± 2 °C and 20 ± 1 °C for four weeks, respectively [23,29]. Wet sifting was used to distinguish immature sclerotia from mature Ss and Rs sclerotia, followed by drying at room temperature. In a pot without plants or seeds, approximately 100 g of soil was mixed separately with 2.5 g of Rs and Ss sclerotia. The pots were then kept in a growth chamber with a day/night photoperiod of 12 h/12 h at 28 ± 2 °C for three months. The soil moisture level was maintained at 60–80% water-filled pore spaces (WFPS) during the study period as performed earlier [42]. Soil without Rs and Ss sclerotia served as a control. The experiment was carried out in triplicate and 10 g of soil was taken from each replicated pot until the third month, and the samples were kept at −80 °C for later use. The soil samples without sclerotia (control) for the 1st, 2nd, and 3rd months are denoted as M1C, M2C, M3C, respectively. Similarly, the soil samples treated with Ss sclerotia for the study period are denoted as M1Ss, M2Ss, and M3Ss, while M1Rs, M2Rs, and M3Rs denote Rs sclerotium treated soil samples.

### 2.2. Amplification of Soil gDNA and Sequencing

Sclerotia were carefully removed from Rs- and Ss-treated soil samples with sterilized forceps to ensure the soil did not contain any sclerotia or their remnants. Approximately 1 g of sclerotium free soil was used for genomic DNA extraction with the HiPure Soil DNA Mini Kit (Magen, Guangzhou, China) in accordance with the manufacturer’s instructions. DNA was subsequently quantified using the Qubit 2.0 Fluorometer (Invitrogen, Carlsbad, CA, USA). V3-V4 hypervariable regions of the 16S ribosomal RNA of bacteria were amplified using “CCTACGGRRBGCASCAGKVRVGAAT” and “GGACTACNVGGGTWTCTAATCC” forward and reverse primers, respectively [43,44]. Moreover, the internal transcribed spacer-2 (ITS2) region was amplified using “GTGAATCATCGARTC” and “TCCTCCGCTTATTGAT” forward and reverse primers to obtain fungal communities [45]. The ITS2 region was preferred over ITS1 due to less taxonomic bias and lower length variation with universal primer sites [46,47]. Additionally, 16S rRNA and ITS2 primers were added to indexed adapter sequences to ensure consistent library amplification. The detailed procedure of 16S rRNA and ITS library amplification and PCR conditions is provided in the Supplementary File. DNA libraries were prepared in the manner as previously mentioned [48]. The Agilent 2100 Bioanalyzer (Agilent Technologies, Palo Alto, CA, USA) and Qubit 3.0 Fluorometer were used to confirm the quantity and quality of the DNA libraries, respectively. The Illumina MiSeq (Illumina, San Diego, CA, USA) platform at GENEWIZ, Inc. (Suzhou, China) was loaded with multiplexed DNA libraries by following the manufacturer’s instructions. MiSeq Control Software v4.0 was used for base calling and image analysis. The sequenced data of ITS2 (control samples, Ss, and Rs treated soil samples) and 16S rRNA (control samples and Ss treated soil samples) have been submitted to NCBI SRA with accession No. PRJNA1288191. The sequenced data of 16S rRNA of Rs sclerotium treated soil samples (PRJNA1288175) was used from our previous study, which was carried out with the same conditions and is included for visual and qualitative comparison only [23].

### 2.3. Downstream Analysis

The sequencing platform provided raw data with adapter sequences and low-quality reads. To initiate the primary analysis, adapter sequences and low-quality reads were trimmed using Cutadapt v1.9.1 [49] to achieve clean reads as previously discussed [50]. The *make.contigs* command in mothur v 1.39.5 [51] was used to pair the clean reads of each sample. Furthermore, clean reads longer than 225 bp and with a minimum score of Q30 were retained, and reads with homopolymer > 8 bases were removed [52]. The paired-end reads were aligned to the Silva reference database (v132) [53,54,55], and reads that failed to align were eliminated [53,56,57]. Correctly aligned effective reads were dereplicated and clustered using the Single Linkage Preclustering technique as described by others [58].

The chimeric sequences were identified and removed by comparing with the Gold database using the UCHIME algorithm [59,60], and the silva database with the Ribosomal Database Project (RDP) (v19) [61,62] classifier was used for 16S rRNA data. Only non-chimeric sequences were subjected to taxonomic assignments. The sequences that failed to classify, or matched mitochondria, chloroplast, or unknown were eliminated. Later, pairwise distances between sequences were calculated to build a distance matrix. Additionally, a majority consensus taxonomy with a 97% similarity threshold was assigned to Operational Taxonomic Units (OTUs) using the average neighbor clustering approach [63]. To prevent sequencing artifacts, singletons were eliminated from the data, and the sample data was rarefied based on the sequences in the smallest samples [54].

PIPITS pipeline was utilized to process raw reads in the case of ITS data with default parameters as explained earlier [64]. Briefly, the raw paired-end reads were cleaned, merged, chimera checked, and clustered into OTUs at 97% similarity. OTUs with single sequences were also removed. Using the UNITE 10.0 database (version 19.02.2025) and an RDP classifier, OTUs were taxonomically assigned [61] with an RDP confidence threshold of 85% and an identity threshold of 97% [65]. Before further analysis, the sample sequences were rarefied to 12179 and 12793 in the case of bacterial and fungal communities, respectively. Good’s coverage scores and diversity indices like number of observed OTUs (OTU richness), Shannon’s diversity, and Pielou’s evenness were estimated using mothur with 10,000 iterations [66].

### 2.4. Statistical Analysis

The data was statistically analyzed using R version 4.5.0 [67]. The Shapiro–Wilk test was employed to confirm the data normality, and the Kruskal–Wallis test determined the differences in the relative abundances of microbial communities. Additionally, significant variations in OTU richness, Pielou’s evenness, and Shannon’s diversity parameters in response to the sclerotial amendment were tested using ANOVA followed by Tukey’s honest significant difference (HSD) test for post hoc comparisons on normally distributed data. Non-normal data was subjected to Kruskal–Wallis test followed by post hoc Dunn’s test with Benjamini–Hochberg correction for multiple comparisons to identify significantly different groups [68]. Bray–Curtis dissimilarity matrices were used for principal coordinate analysis (PCoA) and hierarchical clustering in mothur, followed by visualization in R with ggplot2 version 4.0.0 and Mega 12, respectively [69,70]. The combined panels and legends of the PCoA plot were assembled using cowplot version 1.2.0 and gridExtra version 2.3 packages [71,72].

Permutational analysis of variance (PERMANOVA) was used to examine the varying microbial communities using the built-in functions of the vegan package (version 2.7-1), like vegdist and adonis2, with 10,000 iterations [73]. Additionally, the homogeneity of variances in these dissimilarity metrics was confirmed using the betadisper function. The IndicSpecies package was employed for multipattern analysis of bacterial and fungal OTUs that were strongly linked to Ss and Rs treated and control soil samples [74]. The R package pheatmap (version 1.0.8) was used to perform community cluster analysis using Euclidean distance to determine the differences in bacterial and fungal communities between and within the control, Ss, and Rs treated soil samples [75].

## 3. Results

### 3.1. Overview of the Sequencing Data Quality

A total of 1,630,827 clean paired end reads were obtained from 16S rRNA sequenced data, where the lowest number of reads was 40,132, while the highest number of clean reads was 109,376, with an average number of 60,401 clean reads. Similarly, the number of clean, quality filtered paired end reads obtained was 1,660,386 in the case of ITS2 sequencing. The lowest and highest numbers of paired end reads were 36,624 and 97,581, respectively, with 61,496 being the average number of paired end reads.

### 3.2. Alpha Diversity Matrices of Microbial Communities

**Figure 1:**
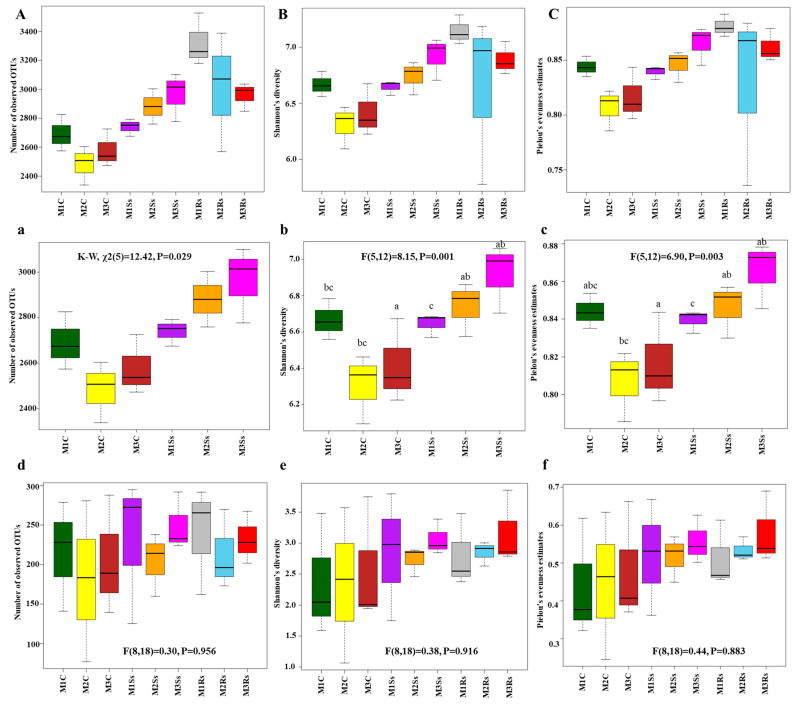
Alpha diversity indices, i.e., observed OTUs (**a**,**d**), Shannon’s Diversity (**b**,**e**), and Pielou’s Evenness Estimates (**c**,**f**) of microbial communities. These were obtained after 16S rRNA (**a**–**c**) and ITS (**d**–**f**) sequencing of control (C), *Sclerotinia sclerotiorum* (Ss) sclerotia treated, and *Rhizoctonia solani* (Rs) sclerotia treated soil samples incubated for three consecutive months (M). Alpha diversity indices, including observed OTUs (**A**), Shannon’s diversity (**B**), and Pielou’s evenness estimates (**C**) of bacterial communities, are also shown for reference, compared with already published data. Effects of control and treated soil samples (F (dfn, dfd) and *p*-value) are indicated in the graphs, while lowercase letters within the graphs represent statistically significant differences (*p* < 0.05) when ANOVA followed by Tukey’s HSD test was performed on normally distributed data.

Before calculating alpha diversity indices, we standardized the OTU tables based on 12179 and 12793 sequences in the case of bacterial and fungal communities, respectively, and removed OTUs containing single sequences to minimize the sequencing artifacts. Good’s coverage scores of 16S rRNA ranged from 84.86 to 88.56%, while for ITS2, it ranged from 99.41 to 99.60%. The data of Shannon’s diversity and Pielou’s evenness estimates was normally distributed, while data of observed OTUs was non-normal, as observed using the Shapiro–Wilk normality test. ANOVA followed by Tukey’s HSD test confirmed statistically significant differences in Shannon’s diversity (*df* = 5, F = 8.15, *p =* 0.001) and Pielou’s evenness estimates (*df* = 5, F = 6.90, *p =* 0.003) in control and Ss treated soil samples. While it was non-significant. The data of observed OTUs (*df* = 5, χ^2^ = 12.42, *p =* 0.028) was non-normal; therefore, the Kruskal–Wallis rank sum test was performed, and it showed a significant difference. Subsequently, the data of Pielou’s evenness estimates was subjected to post hoc Dunn’s test to find out the groups that were different in our data. There were zero and four groups that showed significance with and without *p*-value adjustment using the Benjamini–Hochberg method, respectively (Supporting Information File S1: Table S1). Generally, the OTU richness in controls was around 2575–3260 OTUs (Figure 1a), while OTU richness in Ss-treated soil samples was around 2475–3100 (Figure 1a). The diversity of bacterial communities in control samples varied across the 3 month study period, whereas the diversity of Ss-treated soil samples gradually increased when compared with Rs-treated samples during the same period (Figure 1b,B). Similarly, the evenness was higher in the Ss-treated samples compared to the control (Figure 1c).

In the case of ITS2, alpha diversity indices were normally distributed, as observed using the Shapiro–Wilk test. Non-significant results were observed in the case of OTU richness when one-way ANOVA was performed (*df* = 8, F = 0.30, *p =* 0.956). Shannon’s diversity index and Pielou’s evenness estimates also depicted non-significant results (*df* = 8, F = 0.38, *p =* 0.916 and *df* = 8, F = 0.44, *p =* 0.883, respectively). Generally, the OTU richness of non-amended controls was around 75–280 OTUs, while there were 125–290 and 160–290 OTUs, respectively, in Ss-treated and Rs-treated soil samples (Figure 1d). The diversity of fungal communities in control and treated soil samples varied across the 3 month study period, whereas the diversity of Rs-treated soil samples gradually increased, while Ss-treated soil samples depicted varied responses during the same period (Figure 1e). Similarly, the evenness was higher in Ss- and Rs-treated soil samples compared to the control (Figure 1f).

### 3.3. Beta Diversity

**Figure 2:**
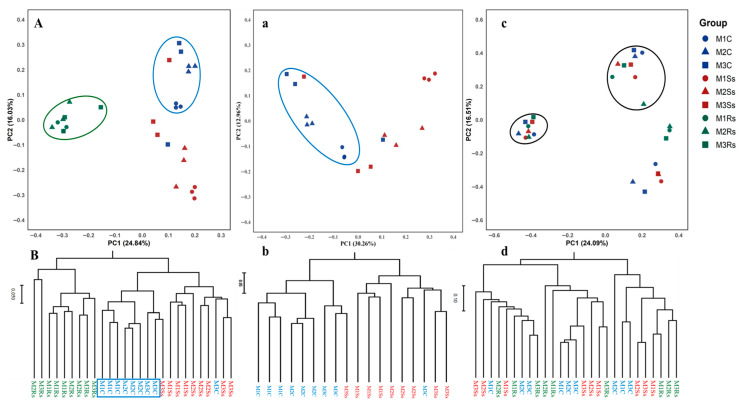
Principal coordinate analysis (PCoA) and hierarchical clustering of bacterial (**a**,**b**) and fungal (**c**,**d**) communities in control (C), *Sclerotinia sclerotiorum* (Ss) sclerotia treated, and *Rhizoctonia solani* (Rs) sclerotia treated soil samples incubated for three consecutive months (M). The blue color represents control samples, while the red and green colors show Ss sclerotia treated and Rs sclerotia treated soil samples, respectively. Circles, triangles, and squares represent the 1st, 2nd, and 3rd months for control, Ss sclerotia treated, and Rs sclerotia treated soil samples. PCoA (**A**) and hierarchical clustering (**B**) compare control and Ss-treated soil samples with our published bacterial community data from Rs-treated soil samples.

Bray–Curtis dissimilarity matrix with rarefied data was used to identify the main drivers of microbial composition in control, Ss, and Rs treated samples (Figure 2). After rarefying the data, 21891 bacterial OTUs were obtained in all samples without singletons. Hierarchical clustering was performed to check the month-wise differences in microbial communities, followed by visualization of samples carried out using Principal Coordinate Analysis (PCoA). PC1 contributed 30.26%, while PC2 explained 12.96% variation in the case of bacterial communities in control and Ss treated soil samples (Figure 2a). Ss-treated and control samples were scattered. The same grouping was also observed in hierarchical clustering of control and treated samples, where M3C and M3Ss were clustered together (Figure 2b).

To clearly find out the differences in bacterial communities, OTUs with cumulative abundance ≤10 in all samples were discarded and subjected to permanova analysis. Permanova of different treatments (F = 4.618, *p* = 0.0001) and months (F = 2.549, *p* = 0.001) showed significant difference, as well as their interaction effect was also significant (F = 1.999, *p* = 0.01), as given in Table 1. The pairwise permanova of control and Ss treated soil samples shows significance with and without fdr adjustments (Supporting Information File S1: Table S2). Moreover, the month-wise effect was also confirmed with pairwise permanova, and we found a statistically significant difference in M1 vs. M3, as shown in Supporting Information File S1: Table S3. Moreover, dispersion tests revealed non-significant differences in within-group variability for treatment (F = 0.95, *p* > 0.05) and month (F = 0.76, *p* > 0.05), ensuring that the significant permanova results are due to sclerotial treatment and are not affected by uneven variances.

Contrary, fungal communities did not reveal clear cut differences in control and treated soil samples as shown in the case of bacterial communities. PC1 revealed 24.01% and PC2 displayed 16.51% variation in fungal communities. We observed two distinct clusters showing control and treated samples, and the same pattern was observed with hierarchical clustering (Figure 2c,d). In the case of fungal communities, Permanova results of different treatments (F = 13.60, *p* = 0.0001) and months (F = 1.53, *p* < 0.05) showed significant difference, while their interaction effect was non-significant (F = 1.234, *p* > 0.05), as given in Table 2. The pairwise permanova of different treatments (control, Ss, and Rs treated soil samples) shows significant differences with and without fdr adjustments, while the pairwise permanova of different months did not show significant differences (Supporting Information File S1: Tables S4 and S5). Dispersion tests revealed non-significant differences in within-group variability for treatment (F = 1.27, *p* > 0.05) and months (F = 1.63, *p* > 0.05), ensuring that the permanova results are not affected by uneven variances.

### 3.4. Phyla Level Shift in Microbial Diversity

**Figure 3:**
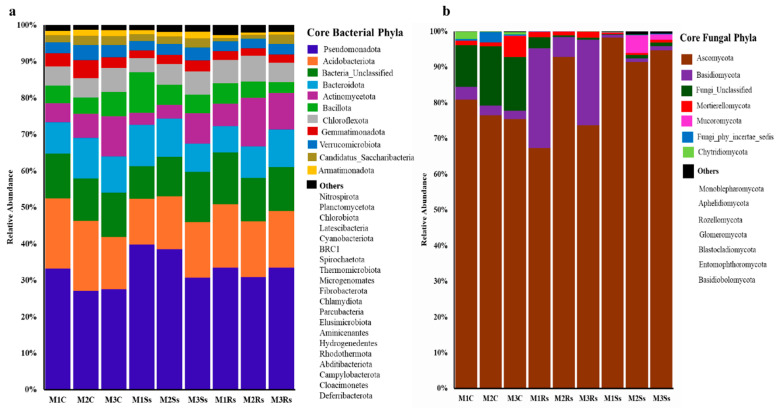
Relative abundance (%) of core bacterial (**a**) and fungal (**b**) phyla depicting increase or decrease in control (C), *Sclerotinia sclerotiorum* (Ss) sclerotia treated, and *Rhizoctonia sloani* (Rs) sclerotia treated soil samples incubated for 3 consecutive months (M). Relative abundance was calculated from high quality sequencing reads assigned to each phylum.

A marked shift in microbial diversity at the phyla level has been observed in control and Ss treated soil samples. About 31 different bacterial phyla were obtained in control and Ss treated soil samples across 3 months. Of these, 11 were categorized into core bacterial phyla as they contributed 97.23–98.70% of the total relative abundance. The remaining 20 phyla only contributed 1.30–2.77% in total relative abundance of control and treated soil samples and were categorized into “Others” (Figure 3a and Supporting Information File S1: Table S6). Specifically, the relative abundance of phylum Pseudomonadota was maximum (30.68–39.80%) in Ss-treated soil samples compared to 30.87–33.54% in Rs-treated samples. While less relative abundance (27.05–33.23%) was observed in non-amended controls. In Acidobacteriota, the trend was different with less relative abundance, ranging from 12.58 to 15.26% in Ss treated soil samples compared to the controls with 14.29–19.32% (Figure 3a and Supporting Information File S1: Table S6).

Similarly, fungal communities also showed a change in their relative abundances in control, Ss, and Rs treated soil samples across three months. Fungal communities belonging to 14 distinct fungal phyla were present in control and treated soil samples. Of these, 7 contributed 99.03–99.97% in all the samples, while the remaining seven showed a relative abundance range from 0.03 to 0.97%. The fungal communities that failed to classify at the phyla level were categorized into Fungi_Unclassified and Fungi_phy_incertae_sedis. Precisely, the highest relative abundance of Ascomycota was evident in Ss treated soil samples, ranging from 91.40% to 98.22%, followed by 67.25–92.82% in Rs treated soil samples, while 75.43–80.83% was attained in controls. The amendment of Ss sclerotia increased the overall relative abundance of Ascomycota during the 3-month period. On the contrary, the amendment of Rs enriched the members of Basidiomycota that ranged from 5.54% to 27.99% compared to Ss treated and control samples. Whereas the relative abundance of Fungi_Unclassified and Mortierellomycota was higher in controls compared to the treated ones (Figure 3b and Supporting Information File S1: Table S7).

### 3.5. Genus Level Shift in Microbial Diversity

**Figure 4:**
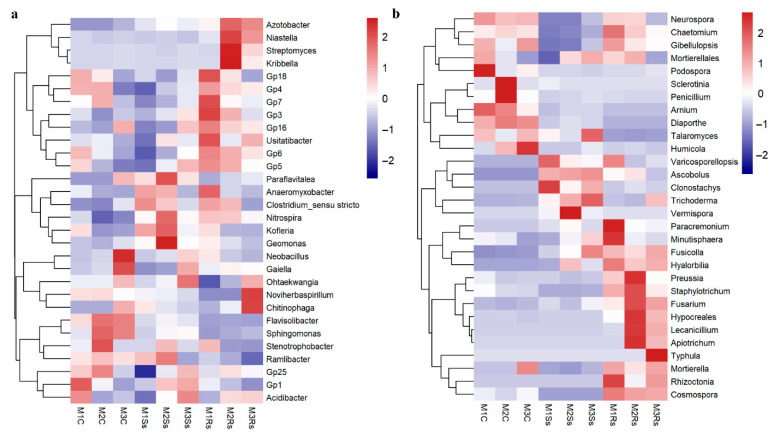
Enrichment/depletion of top 30 bacterial (**a**) and fungal (**b**) genera depicted by Euclidean distance in control (C), *Sclerotinia sclerotiorum* (Ss) sclerotia treated, and *Rhizoctonia sloani* (Rs) sclerotia treated soil samples incubated for 3 consecutive months (M).

We observed a marked shift in microbial diversity at the genus level. To elucidate the effects of control and treated soil samples, we selected the top 30 bacterial genera and denoted them within a heatmap by a Euclidean distance. These selected bacterial genera contributed 38.61–44.43% of relative abundance in controls. While in Ss amended soil samples, these genera attained relative abundance ranging from 27.79% to 33.87%, compared to Rs amended soil samples with 30.46–31.71% relative abundance, respectively (Figure 4a and Supporting Information File S1: Table S8). Precisely, *Acidibacter*, *Stenotrophobacter*, *Sphingomonas*, *Flavisolibacter*, *Gaiella*, and *Neobacillus* depicted enrichment in control samples. On the contrary, *Ramlibacter*, *Geomonas*, *Kofleria*, *Nitrospira*, and *Paraflavitalea* exhibited enrichment in Ss treated soil samples. While *Chitinophaga*, *Noviherbaspirillum*, *Anaeromyxobacter*, *Kribbella*, *Streptomyces*, *Niastella*, and *Azotobacter* showed enhanced abundance in already published data of Rs treated soil samples (Figure 4a and Supporting Information File S1: Table S8).

We subjected the top 30 fungal genera to heatmap based differentiation to elucidate the enrichment/depletion of specific genera in control and treatment samples. These fungal genera contributed 26.02–47.72% of relative abundance in non-amended controls, while in Ss and Rs amended soil samples, the relative abundance ranged from 80.64% to 89.37% and 86.00–93.40%, respectively (Figure 4b and Supporting Information File S1: Table S9). The control samples across the 3 month study period depicted higher abundance of *Mortierella*, *Humicola*, *Diaporthe*, *Arnium*, *Penicillium*, *Sclerotinia*, and *Podospora*, while *Vemispora*, *Trichoderma*, *Clonostachys*, *Ascobolus*, and *Talaromyces* exhibited enrichment in Ss treated soil samples. Moreover, Rs treated soil samples were abundant with *Rhizoctonia*, *Typhula*, *Mortierella*, *Apiotrichum*, *Lecanicillium*, *Fusarium*, *Staphylotrichum*, *Preussia*, *Minutisphaera*, and *Paracremonium* across the three-month study period (Figure 4b and Supporting Information File S1: Table S9). It was noteworthy that various pathogenic genera were more abundant in control samples, such as *Mortierella*, *Humicola*, *Diaporthe*, *Arnium*, *Penicillium*, *Sclerotinia*, and *Podospora* (Figure 4a and Supporting Information File S1: Table S8). On the contrary, *Clonostachys*, *Ascobolus*, *Vermispora*, *Trichoderma*, *Talaromyces*, and *Minutisphaera*, which are reported to play a beneficial role, showed enrichment in treated soil samples, while the notorious pathogenic potential like *Fusarium* was more abundant in treated soil samples (Figure 4b and Supporting Information File S1: Table S8).

### 3.6. Indicator Species Analysis

To further confirm the differentiation of microbial communities between control and treated samples, we performed indicator species analysis and found that there was a significant association between treatment and OTUs. From the top 200 OTUs, we obtained 23 bacterial indicator species, and 6 of these were indicator species of controls, while the remaining 17 were indicator species of Ss- and Rs-treated soil samples. Most of the bacterial genera enriched in Ss and Rs amended samples are of reported beneficial nature, like *Ramlibacter*, *Devoisa*, *Anaeromyxobacter*, *Lysobacter*, *Thiobacillus*, and *Kribbella*, etc. (Table 3).

We only selected indicator species that were assigned to the genus level and found that there were 22 fungal indicator species in our sequenced data. Of these, 12 were indicator species of control samples, while the remaining 10 were indicator species of Ss and Rs treated samples (Table 4). Most of the indicator species of control are reported plant pathogens with few exceptions, while indicator species of treated samples are reported to have beneficial biocontrol properties against several plant pathogenic species.

### 3.7. Distribution of OTUs Across the Months

**Figure 5:**
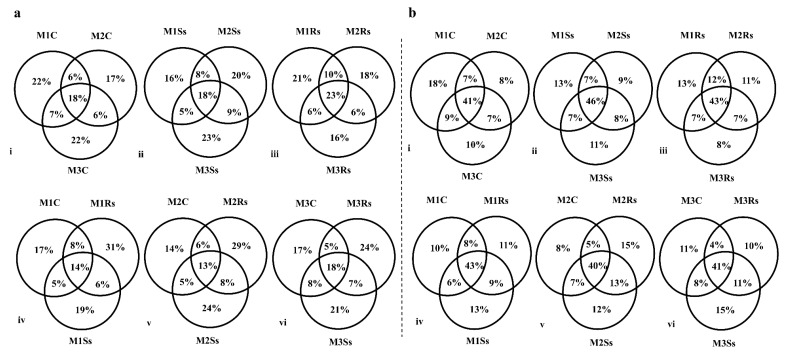
Month-wise percentage of unique and shared OTUs in control (C), Sclerotinia sclerotiorum (Ss) sclerotia treated, and Rhizoctonia solani (Rs) sclerotia treated soil samples incubated for 3 consecutive months (M) in the case of 16S rRNA (**a**) and ITS2 (**b**) sequencing.

The distribution of OTUs in control, Ss, and Rs treated sequenced soil samples was observed during the 3 month study period using a Venn diagram (Figure 5). For this purpose, different combinations were used to know the distribution of OTUs. The unique OTUs in non-amended controls were 22% during the first and third months while 23% and 21% unique OTUs were evident in M3Ss and M1Rs, respectively (Figure 5a(i–iii)). Rs amended samples exhibited the highest percent of unique OTUs, 31%, 29%, and 24%, during M1, M2, and M3, respectively (Figure 5a(iv–vi)). In the case of fungal communities, the percentage of shared OTUs was higher, i.e., 41%, 46%, and 43% across the three months, compared to unique OTUs. A varied response in unique OTUs percentage was evident in control and Ss treated soil samples, while Rs treated samples represented a gradual decline (Figure 5b(i–iii)). When these samples were compared in combination, M1Ss, M2Rs, and M3Ss revealed 13%, 15%, and 15% unique OTUs, respectively (Figure 5b(iv–vi)).

## 4. Discussion

Fungal pathogens are of prime importance because of their contribution, i.e., up to 14% crop losses worldwide annually, making them one of the most important threats to sustainable agriculture and global food security. In addition, some fungi produce survival structures (sclerotia) during unfavorable environmental conditions. These sclerotia help them survive for longer periods in the absence of a host, water, and nutrients and are difficult to manage, being resistant to fungicide application as well. These sclerotia are rich sources of minerals like Mg, K, Ca, Na, along with dietary fibers, fats, chitin, carbohydrates, and proteins. In our study, we mimic the field conditions in the incubator that not only incited the sclerotia but also other soil-residing microbes.

Collectively, we subjected 3.2 million reads to sequencing analysis. Due to the variations in samples, the OTU data were standardized based on the minimum sequences in the data before proceeding to the alpha and beta diversity estimations [76,77]. Resultantly, we obtained 84–88% Good’s coverage score for 16S rRNA and 99% coverage in ITS data. The same results are consistent with our previous findings where the sequencing data of 16S rRNA and ITS2 obtained from rapeseed-fallow field soil samples amended with *Sclerotinia sclerotiorum* depicted 84–91% and 99% Good’s coverage scores, respectively [29]. We obtained 2475–3260 OTUs in control and treated soil samples in the case of 16S rRNA, while OTUs ranged from 75 to 290 in the case of ITS sequencing. These results are consistent with the earlier findings where OTUs range from 950 to 4100 in the soil samples amended with different concentrations of *R. solani* sclerotia and subjected to 16S rRNA sequencing [23]. Findings of other studies where the 16S rRNA sequencing yielded fewer OTUs, i.e., 400–950, while more OTUs, i.e., 230–850, in the case of ITS sequencing, are contrary to our results. Observed OTUs were less in control compared to the treated soil samples. A similar trend was evident in evenness both in the case of 16S rRNA and ITS sequencing.

Phyla level distribution of bacterial communities in control and treated samples showed affiliation with diverse phyla, while fungal communities were more diverse in the control samples compared to the treated samples. These findings are similar to the earlier research [23,29]. Genus-level microbial community distribution revealed that *Flavisolibacter*, *Gaiella*, *Neobacillus*, and *Sphingomonas* were more abundant in control compared to the treated samples. *Flavisolibacter* and *Sphingomonas* are reported plant growth promoters [78,79,80]. Whereas most of the bacteria such as *Chitinophaga*, *Noviherbaspirillum*, *Geomonas*, *Kofleria*, *Kribella*, *Streptomyces*, *Niastella*, *Azotobacter*, and *Anaeromyxobacter* depicted enrichment in treated soil samples compared to the control and have already been reported beneficial to plant growth promotion and biocontrol agents against different plant pathogens [81,82,83,84,85]. Moreover, the control samples were enriched with *Sclerotinia*, *Penicillium*, *Arnium*, and *Diaporthe*, which are well-known pathogenic fungi [86,87,88,89], while the Ss treated soil samples showed enhanced abundance of several fungi that possess beneficial properties like biocontrol ability to restrict the growth of other pathogenic microbes, notably *Humicola*, *Talaraomyces*, *Trichoderma*, *Vermispora*, *Clonstachys*, and *Ascobolus* [89,90,91,92,93,94]. Similarly, Rs treated soil samples exhibited enrichment of beneficial microbes such as *Lecanicillium*, *Hyalorbia*, *Conocybe*, *Minutisphaera*, *Mortierella*, and *Podospora* where the latter serves as a model organism for researchers [95,96,97,98,99,100]. In this study, we found that several fungi, notably *Rhizoctonia*, *Typhula*, *Apiotrichum*, *Fusarium*, *Paracremonium*, and *Chaetomium*, were abundant in Rs treated soil samples and are reported plant pathogens as described earlier [1,101,102,103,104].

Soil dwelling microbes produce several lytic enzymes to hydrolyze different compounds and minerals. Numerous microorganisms are present in the soil, although fungi are more abundant than bacteria when it comes to exhibiting chitinolytic activity. These enzymes may limit the growth of beneficial and pathogenic microbes. In this study, we incubated soil with Ss and Rs sclerotia to mimic the field conditions. We found that soil-dwelling biocontrol agents like *Trichoderma* and *Talaromyces* increased in abundance, while the abundance of *Sclerotinia* declined in the treated soil samples compared to the control during the study period. The abundance of *Rhizocotnia* in Rs treated soil samples declined during the second month but increased again in the third month. During the same period, several beneficial and pathogenic microbes exhibited abundance in treated samples. Sclerotial contents are complex and difficult to hydrolyze as they remain dormant in the soil for several years. The presence of sclerotia in the soil may serve as a nutritious food that has both qualitative and quantitative effects on the bacterial and fungal communities living there due to the presence of proximates and minerals [30]. We believe that because sclerotia contains chitin, the chitin-hydrolyzing soil microbes become activated and utilize it to grow, ultimately altering the microbial diversity through a variety of advantageous functions. Soil bacteria that can withstand the conditions under which different lytic enzymes are produced exhibit enrichment in sclerotium treated soil samples. Previous studies revealed that *Trichoderma*, a soil-borne biocontrol agent that can parasitize a variety of plant pathogens and pests by taking advantage of chitinases and hydrolase enzymes [105,106]. The potent chitinolytic activity of *Trichoderma* species has been extensively researched [89,107,108,109]. These outcomes helped us to presume that the decline in the abundance of pathogenic microbes in Ss treated soil samples is due to the mycoparasitic activity of *Trichoderma* and *Talaromyces*. These outcomes are comparable to earlier research showing that microbial diversity was changed by chitin-hydrolyzing microorganisms and maybe due to the further breakdown of sclerotial nutrients that were utilized by these microbes [12,13,14,15,110,111,112].

We presumed that as sclerotia germinated, *Trichoderma*, *Talaromyces*, and *Streprtomyces* attacked the germinating fungus and restricted their growth. Earlier research found that organic matter infested with *T. virens* and *Coniothyrium minitans* reduced the carpogenic germination of sclerotia [113]. As reported biocontrol agents of *Sclerotinia*, we discovered in this study that *Trichoderma*, *Talaromyces* [89,114], *Clonostachys* [115], and *Streptomyces* [82] exhibited enrichment in soil samples amended by *Sclerotinia*, which ultimately decreased the abundance of *Sclerotinia* after the first month. It is worth noting that Ss treated samples enhanced different plant pathogens and beneficial microbes compared to the Rs treated soil samples. The reason could be the preference of different microbes and availability of nutrients from *Sclerotinia* and *Rhizoctonia* sclerotia.

## 5. Conclusions

It is evident from the current study that every pathogen lives in a microhabitat, that is suitable for its survival. Any imbalance in the microhabitat either in the form of a change in temperature or moisture, could disrupt the survival of this pathogen along with other beneficial and pathogenic microbes. The findings also provided cues that even though *Trichoderma* and *Talaromyces* were abundant, some pathogenic microbes like *Fusarium*, *Rhizoctonia*, and *Typhula*, etc., survived in their presence. Moreover, it is concluded that by activating *Sclerotinia* through irrigation during the off-season, we can reduce the inoculum pressure in the soil and can easily grow the rapeseed or other crop next season. Whereas, in the case of *Rhizoctonia* infected soil, it is suggested to avoid growing crops susceptible to wilt, root rot, and blight, as pathogens causing these diseases exhibited enrichment in the Rs treated soil. Rice-rapeseed rotation is a widely used cropping pattern in Southern China; therefore, the role of sclerotial contribution to the ecology must be considered carefully.

## Figures and Tables

**Table 1:** Analysis of bacterial communities using permutational ANOVA.

	Effect	Df	Sum of Squares	R^2^	F-Value	*p*-Value
With Rs Data (for reference)	Treatment	2	0.9476	0.3658	8.716	<0.0001
	Month	2	0.2585	0.0998	2.378	<0.001
	Treatment × Month	4	0.4058	0.1567	1.866	<0.001
	Residuals	18	0.9784	0.3777		
Without Rs Data	Treatment	1	0.2512	0.1796	4.618	<0.0001
	Month	2	0.2773	0.1983	2.549	<0.001
	Treatment × Month	2	0.2175	0.1555	1.999	<0.01
	Residuals	12	0.6527	0.4667		

Treatment = control and soil treated with sclerotia.

**Table 2:** Analysis of fungal communities using permutational ANOVA.

Effect	Df	Sum of Squares	R^2^	F-Value	*p*-Value
Treatment	2	2.646	0.511	13.600	<0.0001
Month	2	0.298	0.057	1.529	<0.05
Treatment × Month	4	0.480	0.093	1.234	>0.05
Residuals	18	1.751	0.338		

Treatment = control and soil treated with sclerotia.

**Table 3:** Bacterial indicator species in control and treated soil samples.

Sr. No.	Genus or Higher	Treatment	Indicator Value	*p*-Value
1	Gp1	M1C	0.524	0.006 **
2	*Flavisolibacter*	M2C	0.462	0.031 *
3	Gp25	M2C	0.436	0.008 **
4	Gp4	M2C	0.449	0.046 *
5	*Tellurimicrobium*	M2C	0.492	0.029 *
6	*Mycobacterium*	M3C	0.788	0.003 **
7	*Azonexus*	M1Ss	0.715	0.026 *
8	*Ramlibacter*	M1Ss	0.409	0.047 *
9	*Candidatus_Koribacter*	M2Ss	0.513	0.037 *
10	*Geomonas*	M2Ss	0.697	0.009 **
11	*Kofleria*	M2Ss	0.567	0.020 *
12	*Devosia*	M3Ss	0.49	0.015 *
13	*Fimbriimonas*	M3Ss	0.642	0.003 **
14	*Anaeromyxobacter*	M1Rs	0.553	0.044 *
15	*Clostridium_sensu stricto*	M1Rs	0.57	0.023 *
16	Gp16	M1Rs	0.549	0.011 *
17	Gp6	M1Rs	0.529	0.032 *
18	Gp7	M1Rs	0.577	0.013 *
19	*Lysobacter*	M1Rs	0.777	0.033 *
20	*Thiobacillus*	M1Rs	0.705	0.004 **
21	*Kribbella*	M2Rs	0.883	0.001 **
22	*Niastella*	M2Rs	0.732	0.013 *
23	*Reyranella*	M3Rs	0.551	0.030 *

IndVal function of indicSpecies package in R was used for calculating Dufrene-Legendre indicator species in control (C), *Sclerotinia sclerotiorum* (Ss) sclerotia treated, and *Rhizoctonia sloani* (Rs) sclerotia treated soil samples incubated for 3 consecutive months (M). Significance levels: *p* ≤ 0.01 *, *p* ≤ 0.001 **.

**Table 4:** Fungal indicator species in control and treated soil samples.

Sr. No.	Genus or Higher	Treatment	Indicator Value	*p*-Value
1	*Talaromyces*	M1C	0.626	0.006 **
2	*Podospora*	M1C	0.878	0.017 *
3	*Fusarium*	M1C	0.951	0.009 **
4	*Botryosphaeria*	M1C	0.796	0.010 **
5	*Colletotrichum*	M1C	0.918	0.007 **
6	*Angulomyces*	M1C	0.946	0.006 **
7	*Protocreopsis*	M1C	0.655	0.041 *
8	*Penicillium*	M2C	0.873	0.040 *
9	*Aspergillus*	M2C	0.737	0.042 *
10	*Humicola*	M3C	0.763	0.005 **
11	*Malassezia*	M3C	0.646	0.017 *
12	*Lasiodiplodia*	M3C	0.668	0.025 *
13	*Vermispora*	M2Ss	0.877	0.003 **
14	*Hyalorbilia*	M2Ss	0.884	0.003 **
15	*Trichoderma*	M3Ss	0.69	0.015 *
16	*Talaromyces*	M3Ss	0.562	0.027 *
17	*Paracremonium*	M1Rs	0.784	0.004 **
18	*Minutisphaera*	M1Rs	0.539	0.024 *
19	*Endophragmiella*	M1Rs	0.897	0.028 *
20	*Conocybe*	M1Rs	0.962	0.004 **
21	*Lecanicillium*	M2Rs	0.928	0.022 *
22	*Apiotrichum*	M2Rs	0.828	0.027 *

IndVal function of indicSpecies package in R was used for calculating Dufrene-Legendre indicator species in control (C), *Sclerotinia sclerotiorum* (Ss) sclerotia treated, and *Rhizoctonia sloani* (Rs) sclerotia treated soil samples incubated for 3 consecutive months (M). Significance levels: *p* ≤ 0.01 *, *p* ≤ 0.001 **.

## Data Availability

Sequencing data generated in this study has been deposited in NCBI SRA with accession number PRJNA1288191. All the data analyzed during the current study is included in the main manuscript and its Supplementary Information.

## Supplementary Materials

The following supporting information can be downloaded at: https://www.mdpi.com/article/10.3390/jof11100755/s1, Table S1: Post-Hoc Dunn’s test performed on number of observed OTUs obtained after 16S rRNA sequencing analysis in control and treated soil samples; Table S2: Pairwise permanova (Treatment effect) results of bacterial communities in control and Ss treated soil samples; Table S3: Pairwise permanova (Months effect) results of bacterial communities in control and Ss treated soil samples; Table S4: Pairwise permanova (Treatment effect) results of fungal communities in control and Ss treated soil samples; Table S5: Pairwise permanova (Months effect) results of fungal communities in control and Ss treated soil samples; Table S6: Relative abundance (%) of bacterial phyla in control and treated soil samples; Table S7: Relative abundance (%) of fungal phyla in control and treated soil samples; Table S8: Relative abundance (%) of top 30 bacterial genera in control and treated soil samples; Table S9: Relative abundance (%) of top 30 fungal genera in control and treated soil samples.
