# Supplementary material for: Sclerotia-Mediated Soil Microbiome Modulation in Rice–Rapeseed Cropping Systems
Source: J Fungi (Basel). 2025 Oct 21;11(10):755. doi: 10.3390/jof11100755 (PMC12565633; doi:10.3390/jof11100755)
Supplement: Supplementary file 1 [file jof-11-00755-s001.zip › jof-3893663-supplementary.pdf]

Supporting Information for Mehmood et al 2025

Materials and Methods

Library Preparation

For library preparation, a total reaction volume of 25 µL was prepared, consisting of TransStart Buffer, dNTPs, TransStart Taq DNA Polymerase 2.5 U/µL, 2.5 U/µL pfu (2.5 mM each), primer mix, and 20 ng DNA. Following three minutes of initial denaturation at 94°C, there were twenty-four cycles for 16S rRNA, including 5 seconds at 94°C, 90 seconds of annealing at 57°C, 10 seconds of elongation at 72°C, and finally for 5 minutes at 72°C. In the case of ITS2 amplification, thermal cycling conditions included initial denaturation at 94°C for 5 minutes, 25 cycles of 94°C for 30 seconds, annealing at 57°C for 30 seconds, elongation at 72°C for 30 seconds, and finally at 72°C for 5 minutes.

Results

Table S1. Post-Hoc Dunn's test performed on number of observed OTUs obtained after 16S rRNA sequencing analysis in control and treated soil samples

Sr. No.	Comparison	Z	P.unadj	*P.adj
1	M1C - M1Ss	0.382	0.702	0.810
2	M1C - M2C	1.224	0.221	0.332
3	M1Ss - M2C	1.606	0.108	0.271
4	M1C - M2Ss	1.224	0.221	0.415
5	M1Ss - M2Ss	0.841	0.400	0.500
6	M2C - M2Ss	2.447	0.014	0.072
7	M1C - M3C	0.841	0.400	0.546
8	M1Ss - M3C	1.224	0.221	0.474
9	M2C - M3C	0.382	0.702	0.752
10	M2Ss - M3C	2.065	0.0390	0.146
11	M1C - M3Ss	1.606	0.108	0.325
12	M1Ss - M3Ss	1.224	0.221	0.369
13	M2C - M3Ss	2.829	0.004	0.069
14	M2Ss - M3Ss	0.382	0.702	0.702
15	M3C - M3Ss	2.447	0.014	0.108

*P.adj. was performed with Benjamini-Hochberg method

Soil samples of 1st, 2nd, and 3rd month in control are denoted as M1C, M2C, M3C, while treated with Ss sclerotia are denoted as M1Ss, M2Ss, and M3Ss, respectively.

Table S2. Pairwise permanova (Treatment effect) results of bacterial communities in control and Ss treated soil samples

Groups	pairs	Df	Sum of Squares	F.Model	R^2	P-value (Unadjusted)	Adjusted P-value (fdr)
1	Control vs Ss	1	0.251	3.503	0.179	0.002	0.002*

Table S3. Pairwise permanova (Months effect) results of bacterial communities in control and Ss treated soil samples

Groups	pairs	Df	Sum of Squares	F.Model	R^2	P-value (Unadjusted)	Adjusted P-value (fdr)
1	M1 vs M2	1	0.101	1.288	0.114	0.177	0.177
2	M1 vs M3	1	0.209	2.807	0.219	0.009	0.029*
3	M2 vs M3	1	0.106	1.483	0.129	0.098	0.147

Table S4. Pairwise permanova (Treatment effect) results of fungal communities in control and Ss treated soil samples

Groups	pairs	Df	Sum of Squares	F.Model	R^2	P-value (Unadjusted)	Adjusted P-value (fdr)
1	Control vs Ss	1	1.263	13.689	0.461	0.001	0.001**
2	Control vs Rs	1	1.435	13.114	0.450	0.001	0.001**
3	Ss vs Rs	1	1.271	11.112	0.409	0.001	0.001**

Table S5. Pairwise permanova (Months effect) results of fungal communities in control and Ss treated soil samples

Groups	pairs	Df	Sum of Squares	F.Model	R^2	P-value (Unadjusted)	Adjusted P-value (fdr)
1	M1 vs M2	1	0.146	0.676	0.040	0.732	0.82
2	M1 vs M3	1	0.178	0.937	0.055	0.391	0.82
3	M2 vs M3	1	0.122	0.599	0.036	0.820	0.82

Table S6. Relative abundance (%) of bacterial phyla in control and treated soil samples

**Phylum**	**M1C**	**M2C**	**M3C**	**M1Ss**	**M2Ss**	**M3Ss**	**M1Rs**	**M2Rs**	**M3Rs**
Pseudomonadota	33.23	27.05	27.56	39.80	38.56	30.68	33.54	30.87	33.46
Acidobacteriota	19.25	19.32	14.29	12.58	14.46	15.26	17.24	15.25	15.57
Bacteria_unclassified	12.22	11.49	12.18	8.85	10.84	13.78	14.26	11.97	12.01
Bacteroidota	8.60	11.15	9.90	11.38	10.54	7.80	7.25	8.63	10.30
Actinomycetota	5.31	6.55	11.05	3.35	3.68	8.30	6.10	13.35	9.97
Bacillota	4.80	4.54	6.65	11.14	5.59	5.08	5.66	4.41	3.04
Chloroflexota	5.21	5.37	6.60	3.80	5.65	6.42	6.41	7.10	5.37
Gemmatimonadota	3.61	4.83	2.90	2.13	2.45	2.93	2.40	2.00	2.20
Verrucomicrobiota	3.05	4.13	3.39	2.64	3.13	3.59	2.68	2.66	2.90
Candidatus_Saccharibacteria	1.98	2.65	2.46	1.85	1.96	2.55	0.90	1.09	2.62
Armatimonadota	1.12	1.61	1.59	1.04	1.28	1.86	0.76	0.63	0.70
Nitrospirota	0.38	0.26	0.23	0.45	0.54	0.29	0.59	0.53	0.42
Planctomycetota	0.36	0.33	0.35	0.23	0.24	0.32	0.30	0.17	0.17
Chlorobiota	0.18	0.20	0.25	0.29	0.41	0.25	0.42	0.27	0.24
Latescibacteria	0.10	0.15	0.04	0.04	0.06	0.13	0.42	0.26	0.24
Cyanobacteriota	0.10	0.04	0.08	0.04	0.07	0.06	0.17	0.27	0.31
BRC1	0.11	0.08	0.07	0.13	0.14	0.12	0.19	0.10	0.08
Spirochaetota	0.04	0.02	0.01	0.05	0.07	0.06	0.27	0.09	0.06
Thermomicrobiota	0.07	0.05	0.17	0.02	0.02	0.12	0.06	0.14	0.16
Microgenomates	0.03	0.02	0.04	0.00	0.05	0.09	0.09	0.06	0.04
Fibrobacterota	0.05	0.05	0.07	0.06	0.02	0.04	0.06	0.03	0.03
Chlamydiota	0.02	0.02	0.02	0.03	0.05	0.08	0.04	0.03	0.03
Parcubacteria	0.06	0.00	0.01	0.05	0.08	0.08	0.00	0.00	0.00
Elusimicrobiota	0.04	0.03	0.02	0.06	0.05	0.06	0.03	0.01	0.01
Aminicenantes	0.01	0.00	0.01	0.00	0.00	0.00	0.07	0.03	0.05
Hydrogenedentes	0.01	0.02	0.01	0.01	0.04	0.02	0.01	0.01	0.01
Rhodothermota	0.02	0.02	0.01	0.01	0.00	0.02	0.02	0.02	0.01
Abditibacteriota	0.02	0.01	0.02	0.00	0.01	0.02	0.00	0.00	0.00
Campylobacterota	0.00	0.00	0.00	0.00	0.00	0.00	0.01	0.00	0.00
Cloacimonetes	0.00	0.00	0.00	0.01	0.01	0.00	0.00	0.00	0.00
Deferribacterota	0.00	0.00	0.00	0.00	0.00	0.00	0.01	0.00	0.00

Soil samples of 1st, 2nd, and 3rd month in control are denoted as M1C, M2C, M3C, while treated with Ss and Rs sclerotia are denoted as M1Ss, M2Ss, and M3Ss and M1Rs, M2Rs, and M3Rs, respectively.

Table S7. Relative abundance (%) of fungal phyla in control and treated soil samples

Phylum	M1C	M2C	M3C	M1Ss	M2Ss	M3Ss	M1Rs	M2Rs	M3Rs
Ascomycota	80.83	76.48	75.43	98.22	91.40	94.70	67.25	92.82	73.68
Basidiomycota	3.55	2.69	2.27	0.72	1.00	1.14	27.99	5.54	23.99
Fungi_Unclassified	11.68	16.67	15.11	0.33	0.99	1.00	3.10	0.52	0.53
Mortierellomycota	1.24	0.99	5.84	0.25	0.50	0.81	1.39	1.00	1.61
Mucoromycota	0.03	0.01	0.16	0.11	4.95	1.52	0.06	0.02	0.01
Fungi_phy_Incertae_sedis	0.49	2.92	0.24	0.05	0.16	0.18	0.09	0.04	0.06
Chytridiomycota	2.07	0.17	0.73	0.04	0.03	0.02	0.06	0.03	0.06
Monoblepharomycota	0.03	0.02	0.10	0.23	0.92	0.51	0.01	0.01	0.01
Aphelidiomycota	0.01	0.01	0.06	0.03	0.01	0.12	0.02	0.01	0.02
Rozellomycota	0.02	0.03	0.03	0.00	0.01	0.00	0.01	0.00	0.01
Glomeromycota	0.04	0.00	0.00	0.02	0.02	0.00	0.00	0.00	0.00
Blastocladiomycota	0.00	0.00	0.03	0.00	0.00	0.00	0.01	0.01	0.00
Entomophthoromycota	0.00	0.00	0.00	0.00	0.00	0.00	0.00	0.00	0.01
Basidiobolomycota	0.00	0.00	0.00	0.00	0.00	0.00	0.00	0.00	0.01

Soil samples of 1st, 2nd, and 3rd month in control are denoted as M1C, M2C, M3C, while treated with Ss and Rs sclerotia are denoted as M1Ss, M2Ss, and M3Ss and M1Rs, M2Rs, and M3Rs, respectively.

Table S8. Relative abundance (%) of top 30 bacterial genera in control and treated soil samples

Genus	M1C	M2C	M3C	M1Ss	M2Ss	M3Ss	M1Rs	M2Rs	M3Rs
Flavisolibacter	8.07	14.44	11.39	4.07	4.49	3.99	0.90	1.08	1.17
Gp6	6.33	4.71	2.83	1.90	2.84	4.25	5.55	4.80	4.78
Gp4	4.09	4.36	1.48	1.37	2.19	2.51	3.24	2.63	2.94
Gp7	2.12	3.24	1.24	0.82	1.11	1.61	3.12	1.59	1.65
Gp3	1.64	1.26	1.35	1.37	1.44	1.75	2.22	1.69	1.42
Sphingomonas	1.48	4.04	3.10	1.27	1.50	1.69	0.50	0.62	0.91
Neobacillus	0.97	1.28	4.19	1.09	1.13	2.13	1.54	0.77	0.68
Stenotrophobacter	1.34	2.06	1.11	1.25	1.39	1.11	1.20	0.79	0.93
Kofleria	1.43	0.57	0.53	1.85	2.00	0.98	1.05	0.53	0.58
Gp1	2.13	1.13	0.57	0.87	1.31	1.45	0.78	0.60	0.49
Anaeromyxobacter	0.91	0.25	0.19	2.21	1.76	0.32	2.14	0.49	0.37
Paraflavitalea	0.00	0.00	1.70	1.49	2.72	1.22	0.09	0.42	0.71
Gp16	0.58	0.47	1.34	0.25	0.36	1.20	1.33	0.93	0.99
Clostridium_sensu stricto	0.43	0.39	0.73	1.65	1.12	0.68	1.14	1.00	0.44
Streptomyces	0.20	0.17	0.27	0.15	0.10	0.21	0.12	3.87	1.60
Gaiella	0.57	0.72	2.01	0.20	0.26	1.40	0.62	0.76	0.81
Niastella	0.25	0.17	0.19	0.17	0.18	0.12	0.32	2.10	1.70
Ohtaekwangia	0.64	0.67	0.86	0.55	0.57	1.10	0.11	0.32	0.89
Noviherbaspirillum	0.90	0.98	0.60	0.62	0.51	0.41	0.02	0.02	1.68
Nitrospira	0.54	0.17	0.25	0.70	1.06	0.60	0.66	0.63	0.56
Chitinophaga	0.01	0.01	1.24	0.87	0.38	0.26	0.04	0.38	2.01
Geomonas	0.19	0.02	0.33	0.70	2.20	0.57	0.56	0.26	0.13
Usitatibacter	0.57	0.63	0.40	0.33	0.61	0.51	0.77	0.42	0.72
Acidibacter	0.83	0.37	0.37	0.25	0.47	0.73	0.28	0.46	0.58
Ramlibacter	0.63	0.78	0.57	0.73	0.80	0.28	0.31	0.24	0.13
Gp25	0.59	0.74	0.39	0.22	0.41	0.54	0.34	0.41	0.43
Gp18	0.62	0.52	0.25	0.38	0.24	0.35	0.59	0.37	0.26
Kribbella	0.01	0.00	0.04	0.01	0.01	0.02	0.04	2.26	0.60
Gp5	0.54	0.30	0.15	0.15	0.35	0.53	0.52	0.46	0.38
Azotobacter	0.00	0.00	0.10	0.30	0.35	0.30	0.34	0.81	0.82

Soil samples of 1st, 2nd, and 3rd month in control are denoted as M1C, M2C, M3C, while treated with Ss and Rs sclerotia are denoted as M1Ss, M2Ss, and M3Ss and M1Rs, M2Rs, and M3Rs, respectively.

Table S9. Relative abundance (%) of top 30 fungal genera in control and treated soil samples

Genus	M1C	M2C	M3C	M1Ss	M2Ss	M3Ss	M1Rs	M2Rs	M3Rs
Ascobolus	0.00	0.01	0.44	22.42	26.40	28.11	7.34	8.65	3.89
Hypocreales	0.62	0.14	0.15	0.18	0.37	0.13	6.00	34.41	21.27
Paracremonium	0.00	0.08	0.02	3.95	11.69	3.02	29.02	5.07	5.31
Rhizoctonia	0.10	0.04	0.11	0.00	0.00	0.01	26.89	4.14	21.71
Fusarium	4.21	3.50	8.29	5.62	4.14	8.20	5.64	12.63	11.88
Clonostachys	0.48	0.38	0.59	29.42	8.75	17.65	0.81	1.92	1.23
Lecanicillium	0.00	0.00	0.00	0.00	0.00	0.00	0.44	19.44	13.67
Talaromyces	9.34	4.30	18.69	4.76	3.10	12.49	0.15	0.09	0.25
Trichoderma	0.29	0.36	1.26	4.09	8.04	11.58	0.62	0.41	4.75
Varicosporellopsis	0.00	0.00	0.00	7.23	2.98	2.17	3.50	0.27	0.92
Vermispora	0.01	0.01	0.01	1.40	14.77	1.29	0.26	0.28	0.35
Arnium	5.71	4.80	3.68	0.05	0.39	0.37	0.03	0.00	0.01
Fusicolla	0.12	0.17	0.45	0.58	1.03	2.13	0.87	0.69	1.22
Mortierella	0.52	0.52	5.18	0.13	0.02	0.26	1.06	0.68	1.36
Preussia	0.63	0.17	0.46	0.17	0.07	0.14	0.73	1.66	0.46
Sclerotinia	0.02	7.06	0.04	0.28	0.05	0.06	0.02	0.00	0.02
Hyalorbilia	0.00	0.00	0.01	0.04	0.89	0.32	0.68	0.42	0.68
Mortierellales	0.68	0.42	0.50	0.12	0.48	0.55	0.25	0.28	0.16
Cosmospora	0.33	0.24	0.71	0.03	0.03	0.04	0.43	0.36	0.45
Neurospora	0.77	0.64	1.26	0.01	0.02	0.17	0.25	0.23	0.11
Podospora	2.17	0.16	0.96	0.03	0.05	0.01	0.04	0.03	0.09
Apiotrichum	0.00	0.00	0.00	0.00	0.00	0.00	0.00	0.92	0.55
Staphylotrichum	0.28	0.16	0.28	0.00	0.00	0.02	0.27	0.43	0.21
Chaetomium	0.32	0.38	0.65	0.02	0.03	0.09	0.26	0.17	0.17
Typhula	0.00	0.00	0.00	0.00	0.00	0.00	0.00	0.00	1.34
Diaporthe	0.51	0.69	1.20	0.06	0.12	0.10	0.00	0.00	0.00
Gibellulopsis	0.36	0.20	0.76	0.00	0.01	0.11	0.18	0.11	0.13
Minutisphaera	0.19	0.17	0.14	0.07	0.16	0.23	0.24	0.08	0.08
Penicillium	0.16	1.02	0.32	0.00	0.01	0.05	0.02	0.01	0.02
Humicola	0.05	0.42	1.57	0.00	0.00	0.08	0.01	0.02	0.00

Soil samples of 1st, 2nd, and 3rd month in control are denoted as M1C, M2C, M3C, while treated with Ss and Rs sclerotia are denoted as M1Ss, M2Ss, and M3Ss and M1Rs, M2Rs, and M3Rs, respectively.
